# Manganese(II) complexes with Bn-tpen as powerful catalysts of cyclohexene oxidation

**DOI:** 10.1007/s11696-017-0201-0

**Published:** 2017-05-26

**Authors:** Katarzyna Rydel-Ciszek, Maria Charczuk, Tomasz Pacześniak, Paweł Chmielarz

**Affiliations:** Department of Physical Chemistry, Faculty of Chemistry, Rzeszów University of Technology, 35-959 Rzeszów, Poland

**Keywords:** Dioxygen activation, Cyclohexene oxidation, Manganese(II) complexes, Bn-tpen transition metal complexes

## Abstract

Manganese(II) complex [(Bn-tpen)Mn^II^]^2+^ activated dioxygen for oxidation of cyclohexene in acetonitrile (MeCN) and methanol (MeOH). In MeCN, ketone (2-cyclohexen-1-one), alcohol (2-cyclohexen-1-ol) and small amounts of epoxide (cyclohexene oxide) were produced in this reaction, while in MeOH only ketone was formed. In the most efficient experiment, the combination of 2.5 × 10^−4^ mol% [(Bn-tpen)Mn^II^]^2+^ and 4 M cyclohexene under dioxygen atmosphere (*p*_O2_ = 1 atm) in MeCN after 24 h of reaction, gave the TON equal to 716, and the main oxidation products were ketone (196 mM) and alcohol (147 mM), whereas epoxide was formed in insignificant amounts (15 mM). The formation of [(Bn-tpen)Mn^IV^=O]^2+^ and [(Bn-tpen)Mn^III^–OH]^2+^ species was confirmed. The novelty of this work is the observation, that in both solvents, [(Bn-tpen)Mn^II^]^2+^ complex is initially oxidized by *t*-BuOOH to produce Mn(III)-complex, which is reduced back by cyclohexene to [(Bn-tpen)Mn^II^]^2+^, and the latter species is an active catalyst of *c*-C_6_H_10_ oxidation. Knowledge of the electrochemical properties of the system components may contribute to understanding the mechanisms involving participation of the active agents created in the system.

**Electronic supplementary material:**

The online version of this article (doi:10.1007/s11696-017-0201-0) contains supplementary material, which is available to authorized users.

## Introduction

**Fig. 1:**
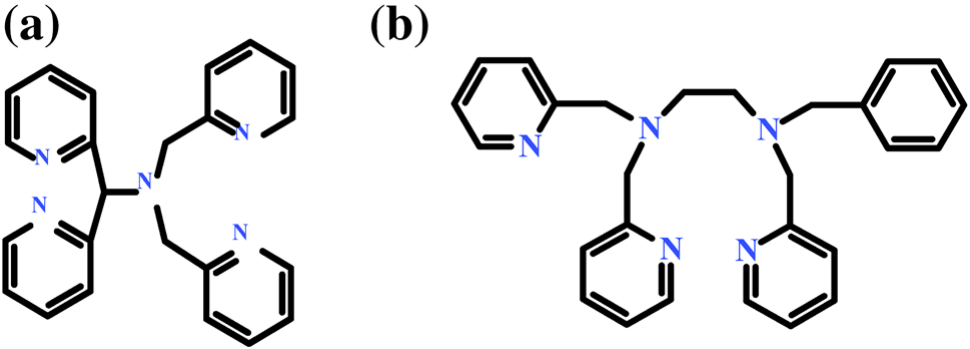
Structures of *N*-pentadentate ligands **a** *N,N*-bis(2-pyridylmethyl)-*N*-(bis-2-pyridylmethyl)amine) (N4Py), **b** *N*-benzyl-*N,N′,N′*-tris(2-pyridylmethyl)-1,2-diaminoetane (Bn-tpen)

Nonheme complexes of iron(II) with *N*-pentadentate ligands like *N,N*-bis(2-pyridylmethyl)-*N*-(bis-2-pyridylmethyl)amine) (N4Py, Fig. 1a) and *N*-benzyl-*N,N′,N′*-tris(2-pyridylmethyl)-1,2-diaminoethane (Bn-tpen, Fig. 1b), and their oxidized iron(IV)-oxo and iron(III)-hydroperoxo forms arouse interest in bioinorganic chemistry (Kaizer et al. 2004; Sahu et al. 2014; Chen et al. 2015; Mitra et al. 2015; Puri et al. 2016).

This kind of solvent influences not only the spin state of [(N4Py)Fe^II^]^2+^ and [(Bn-tpen)Fe^II^]^2+^ complexes, but also their redox potentials and the possibility of the formation of active hydroperoxide species in the presence of dioxygen (Hong et al. 2009). Low-spin iron(II) complexes of lower redox potentials, present in acetonitrile, are not able to activate dioxygen for oxidation of organic substrate, in contrast to high-spin iron(II) complexes, appearing in methanol. In case of oxygen activation by [(N4Py)Fe^II^]^2+^ or [(Bn-tpen)Fe^II^]^2+^ complexes, dioxygen is bound by a high-spin iron(II) complex forming Fe^III^–OO^**·**^ intermediate, which is conversed to a low-spin Fe^III^–OOH species due to proton and electron uptake. Complexes [(N4Py)Fe^II^]^2+^ and [(Bn-tpen)Fe^II^]^2+^ are also oxidized by PhIO to give iron(IV)-oxo species (Costas et al. 2004; Usharani et al. 2013; Wang et al. 2013; Mitra et al. 2015; Barbieri et al. 2016). However, their manganese counterparts were much less studied, because there was a widely held belief that manganese-oxo, and hydroxo- species were less reactive ones (Wu et al. 2011; Leto et al. 2013; Chen et al. 2015). Herein, we claim that Bn-tpen manganese complexes can activate dioxygen, hydrogen peroxide, and *tert*-butyl hydroperoxide for oxidation of unreactive C–H bonds of *c*-C_6_H_10_ in acetonitrile and methanol solutions.

High-valent manganese-oxo intermediates are very important components of artificial manganese clusters for water oxidation, constitute an important part of the oxygen-evolving complex in photosystem II, as well as in oxidation of organic compounds (McEvoy and Brudvig 2006; Wu et al. 2011; Siegbahn 2013; Pham and Messinger 2014; Cox et al. 2015; Young et al. 2016). In search for the method of increasing the oxidizing power of pentadentate high-valent complexes, the influence of a central metal oxidation state, a type of supporting and axial ligands, as well as incorporation of redox-inactive metal ions (playing the role of Lewis acids) to the Mn-oxo moiety on the properties of the complex were analysed (Siegbahn 2009; Chen et al. 2013; Yoon et al. 2013). Theoretical investigations revealed that [(N4Py)Mn^IV^=O]^2+^ is more reactive than [(Bn-tpen)Mn^IV^=O]^2+^ (Cho et al. 2012). It was also reported, that [(Bn-tpen)Mn^IV^=O]^2+^ species participate in oxidation of strong C–H bonds of alkanes like cyclohexane, cyclooctane, ethylbenzene, cumene and others, and proposed that activation of C–H bond by nonheme Mn(IV)-oxo complex does not occur via an oxygen-rebound mechanism (Wu et al. 2011). Some diverse effects of a redox-inactive metal ion (e.g., Sc^3+^) on the reactivities of high valent Mn(IV)-oxo species in oxidation of 9,10-dihydroanthracene and toluene were demonstrated (Chen et al. 2015).

Furthermore, complexes of manganese(II, III) with other ligands like picolinic acid (PA), 2,6-pyridinedicarboxylic acid (DPAH_2_), 2,2′-bipyridine (bpy), triphenylphosphine oxide (OPPh_3_), and tetradentate Schiff-bases in combination with *tert*-butyl hydroperoxide activate dioxygen to oxygenate cyclohexene to ketone, alcohol, and epoxide (Matsushita et al. 1999). The product profiles depend on the ligand and solvent matrix (Matsushita et al. 1999). With PA, bpy, or OPPh_3_ as the ligand, in pyridine/acetic acid (2:1 molar ratio) the dominant product is ketone [*c*-C_6_H_8_(O)], whereas, Schiff-bases complexes produce *c*-C_6_H_8_(O), *c*-C_6_H_9_(OH), and epoxide in almost equal yields. However, in acetonitrile, *c*-C_6_H_8_(O) is the dominant product for all of the complexes (Matsushita et al. 1999).

The above-mentioned findings encouraged us to investigate pentadentate manganese(II) complexes with Bn-tpen as catalysts of the reaction of cyclohexene oxidation by dioxygen and hydroperoxides. Cyclohexene was used as a substrate, because it is very good model compound, it contains a single C–H bond and a double C=C bond, both of which can be an object of oxidation by high-valent manganese complexes and it was proved that it is a good starting point to better understand the mechanism of the reaction of more complex molecular entities, for example, the terpene ozonolysis (Nøjgaard et al. 2007). It is also known, that the selective oxidation of unreactive C–H bonds of organic compounds is a crucial stage of catalysis (Gao et al. 2015; Peng et al. 2016). The exact knowledge of the mechanism of its oxidations may contribute to understanding of the processes occurring in living organisms (e.g., action of enzymes and oxygen toxicity), or in technological processes. It can enable the production of the compounds of high significance in industry (e.g., by the oxidation of aromatic compounds, olefins, alcohols, sulphides, and in reactions of *N*-dealkylation). In this paper we present the novel results of cyclohexene oxidations by dioxygen, HOOH or *t*-BuOOH, activated by [(Bn-tpen)Mn^II^]^2+^, which create potential possibilities of using dioxygen activated manganese(II) complexes as a versatile oxidant in the oxidation of organic compounds.

## Experimental

The solvents for all of the experiments were acetonitrile (≥99.9%, HPLC grade) and methanol (≥99.9%, HPLC grade) purchased from Aldrich. High-purity argon gas was used to deaerate the solutions. Tetraethylammonium perchlorate [(Et_4_N)ClO_4_, 98.0% GFS Chemicals] and tetraethylammonium tetrafluoroborate [(Et_4_N)BF_4_, 99% Fluka] was dried in vacuo over CaSO_4_ for 24 h prior to use. Manganese(II) perchlorate [Mn(ClO_4_)_2_• 6H_2_O, 99%]; hydrogen peroxide, [HOOH, (50% solution in water)]; *tert*-butyl hydroperoxide, [*t*-BuOOH, (70% solution in water or 5.0–8.0 M solution in decane)] and the organic substances: biphenyl (PhPh, ≥99%), cyclohexene (≥99%), 2-cyclohexen-1-one (95%), 2-cyclohexen-1-ol (95%), cyclohexene oxide (98%), were obtained from Aldrich. These reagents were used without further purification.

The reaction products were separated and identified with a Hewlett-Packard 4890A Series gas chromatograph equipped with an HP-1 capillary-column (cross-linked methyl-silicone-gum phase, 30 m × 0.53 mm i.d.).

A three-electrode Autolab potentiostat was used to record the voltammograms. The experiments were conducted in a 2-mL electrochemical cell with provision to control the presence of dioxygen with an argon-purge system. The working electrode was a Bioanalytical Systems glassy-carbon (area, 0.008 cm^2^) inlay, the auxiliary electrode—a platinum wire, and the reference electrode—an Ag/AgCl wire in an aqueous tetramethylammonium chloride solution that was adjusted to give a potential of 0.00 V vs. SCE. The latter was contained in a Pyrex tube with a cracked soft-glass tip, which was placed inside a Luggin capillary (Sawyer et al. 1995).

The UV–vis spectrophotometric measurements were made with a Hewlett-Packard Model HP-8453 diode array rapid scan spectrophotometer.

**Fig. 2:**
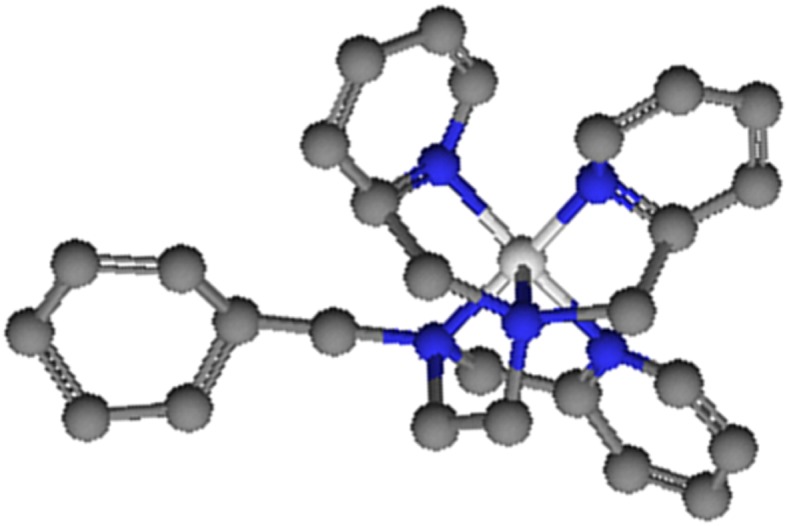
Structure of [(Bn-tpen)Mn^II^]^2+^ catalyst, obtained in CaChe by the use of AM1 procedure

The [(Bn-tpen)Mn^II^]^2+^ catalyst (Fig. 2) was prepared in situ by mixing the stoichiometric amounts of manganese(II) perchlorate and *N*-benzyl-*N,N′,N′*-tris(2-pyridylmethyl)-1,2-diaminoetane in deaerated solvents for 3 h in Ar atmosphere. The Bn-tpen ligand was synthesized according to the procedure given in the literature (Blicke and Maxwell 1939; Roelfes et al. 1997; Duelund et al. 2001). The oxidation peak of the Bn-tpen ligand (+0.85 V), shifts after addition of Mn(ClO_4_)_2_ to the potential +1 V, what indicates the formation of the [(Bn-tpen)Mn^II^]^2+^ complex.

The volume of dioxygen evolved due to hydrogen peroxide decomposition was measured in tightly closed vessel, provided with two necks. The first one was closed by a septum, by which hydrogen peroxide was injected, whereas the second one was connected to a manometric burette. The burette was filled with saturated, aqueous solution of NaCl, saturated with dioxygen before the measurement. The solution was continuously mixed during the analysis and the pressures of the gases inside and outside the vessel were equilibrated, adjusting the position of a separator connected to the second ending of the burette, so as the levels of the liquid inside the burette and the separator were the same. The measured gas volume was corrected for current temperature and pressure using the ideal gas law.

Preparative oxidation and analysis of the products—the appropriate amounts of metal salt and ligand were combined in acetonitrile or in methanol followed by the addition of substrate (cyclohexene) in the reaction cell (25 cm^3^ vial with cut-out cap and Teflon-faced septum, total volume of the reaction mixture was 5 cm^3^). The solution was either saturated with gas oxidant, dioxygen (O_2_, 1 atm) or air (O_2_, 0.2 atm), or with high-purity argon gas with subsequent injection of hydrogen peroxide or *tert*-butyl hydroperoxide as an oxidant. In case of dioxygen and air the specific gas, saturated with acetonitrile or methanol was bubbled through the solution to maintain a constant concentration of oxygen throughout the process. The reactions were allowed to proceed for 24 h with constant stirring at room temperature (23 ± 1 °C). The samples were taken after the experiment and injected into a capillary-column gas chromatograph for analysis. Separately prepared standard samples were always used to confirm the product identifications and to prepare calibration curves for quantitative assays of the product species. Biphenyl (2.5 × 10^−3^ mol%) was used as an internal standard. All experiments were done in triplicate. The presented values of concentration are the mean values of 3 independent experiments.

## Results and discussion

**Fig. 3:**
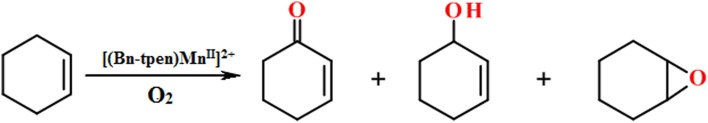
Products of cyclohexene oxidation

In the presence of dioxygen activated by [(Bn-tpen)Mn^II^]^2+^ cyclohexene is oxidized mainly to ketone (2-cyclohexen-1-one), alcohol (2-cyclohexen-1-ol) and epoxide (cyclohexene oxide) (Fig. 3).

**Fig. 4:**
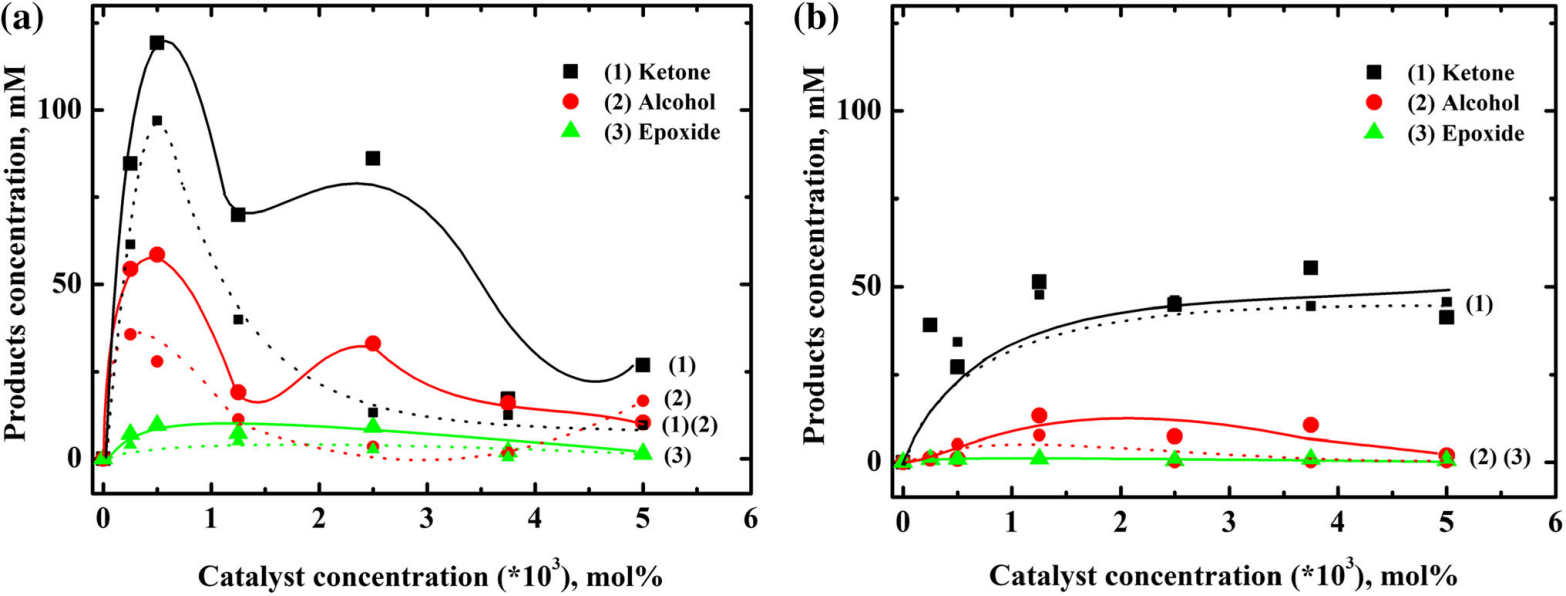
Dependence of products [as: (*1*) 2-cyclohexen-1-one, (*2*) 2-cyclohexen-1-ol, and (*3*) cyclohexene oxide] concentration on catalyst concentration for oxidation of 1 M cyclohexene by dioxygen (*solid lines*) and air (*dot lines*) catalyzed by [(Bn-tpen)Mn^II^]^2+^ in **a** acetonitrile, **b** methanol. Reaction time: 24 h

The results of oxidation reactions for different catalyst concentrations in acetonitrile and methanol are presented in Fig. 4, and Table S-1.

The oxidation of cyclohexene was carried out either using oxygen or air as an oxidant. In MeCN the reaction yields for dioxygen (*p*_O2_ = 1 atm) are higher than those for air (*p*_O2_ = 0.2 atm). The maximum yield is observed for the catalyst concentration between 2.5 × 10^−4^ and 2.5 × 10^−3^ mol%. It is worth to notice, that when the catalyst concentration equals 2.5 × 10^−3^ mol %, the amount of products formed during dioxygen-driven oxidation is much higher than the one obtained using air, and the concentrations of ketone and alcohol are, respectively, approx. 6 and 9 times higher for O_2_ (Fig. 4a, Table S-1). For the catalyst concentrations higher than 2.5 × 10^−3^ mol %, oxidation products are still formed, but the reaction yields are lower. The product profile after 24 h reaction time indicates that the combination of 2.5 × 10^−4^ mol % $\left[ {\left( {\text{Bn - tpen}} \right){\text{Mn}}^{\text{II}} } \right]^{ 2+ }_{\text{MeCN}} /{\text{O}}_{ 2} (p_{\text{O2}} \; = \; 1 {\text{atm}})/ 1 {\text{M}}$ cyclohexene is the most reactive one, with 292 products/catalyst turnovers (Table S-1). When the solvent is changed to MeOH the overall efficiency is reduced (Fig. 4b, Table S-1). Contrary to oxidations in MeCN, virtually the only oxidation product formed in methanol, both in dioxygen and air atmosphere is 2-cyclohexen-1-one. Alcohol and epoxide products are formed in small amounts or are not formed at all. There is no substantial difference in reaction yields when using dioxygen (*p*_O2_ = 1 atm) or air (*p*_O2_ = 0.2 atm) as an oxidant. The increase in reaction yield with the increase in catalyst concentration is not observed in MeOH. The blank experiments conducted in both MeCN and in MeOH show that in the absence of catalyst no products are formed.

**Fig. 5:**
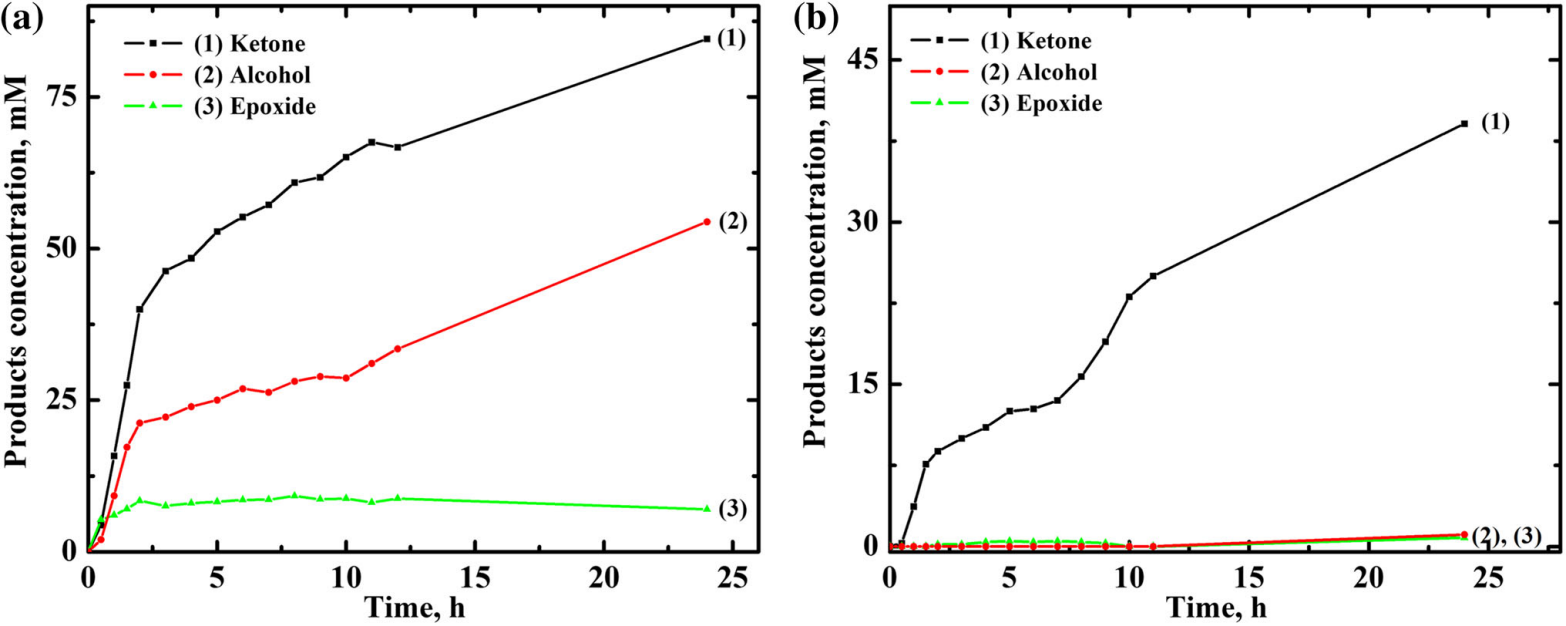
Dependence of products [as: (*1*) 2-cyclohexen-1-one, (*2*) 2-cyclohexen-1-ol, and (*3*) cyclohexene oxide] concentration on time for oxidation of 1 M cyclohexene by dioxygen catalyzed by 2.5 × 10^−4^ mol% [(Bn-tpen)Mn^II^]^2+^ in **a** acetonitrile, **b** methanol

The products profiles suggest that the products are formed independently (Fig. 5). In MeCN (Fig. 5a) almost linear increase in ketone and alcohol concentration during the first 2 h of the reaction is observed. At the beginning the formation of ketone and alcohol is fast—within 2 h almost half of their final (determined after 24 h) concentration is formed. However, the oxidation continues throughout at least 24 h, which indicates that the catalyst is still active. In MeOH, similarly to acetonitrile, the catalyst remains active during the controlled period of 24 h, but in contrast to MeCN, only 2-cyclohexen-1-ol is formed (Fig. 5b) in this solvent.

The dependence of the amounts of formed products on substrate concentration was also investigated (Table S-2). The results show that the increase in the substrate concentration causes almost proportional increase in the reaction molar yield. For substrate concentration from 0.5 to 1 M the amount of ketone formed in acetonitrile is about 2 times higher than one in methanol solutions. For 2.5 × 10^−4^ mol % $\left[ {\left( {\text{Bn - tpen}} \right){\text{Mn}}^{\text{II}} } \right]^{ 2+ }_{\text{MeOH}} /{\text{O}}_{ 2} (0. 2 {\text{atm}})/0. 5 {\text{M}}$ cyclohexene, ketone is the dominant product, whereas for 2.5 × 10^−4^ mol % $\left[ {\left( {\text{Bn - tpen}} \right){\text{Mn}}^{\text{II}} } \right]^{ 2+ }_{\text{MeOH}} /{\text{O}}_{ 2} (0. 2 {\text{atm}})/ 4 {\text{M}}$ cyclohexene system, not only ketone, but also alcohol and epoxide are produced in significant amounts.

In some experiments either HOOH or *t*-BuOOH was added to the reaction mixture as an oxidant. In acetonitrile solutions, both for HOOH and *t*-BuOOH the reaction yields are lower in comparison to air or dioxygen (Table S-3). It is interesting that for the mentioned peroxides alcohol is formed as the main product (Table S-3). When the reactions are carried out in methanol the use of hydrogen peroxide or *tert*-butyl hydroperoxide also gives much smaller yields than ones obtained for dioxygen, and ketone is the only product (Table S-4).

It is characteristic that the reaction mixture rapidly changes colour after introduction of *t*-BuOOH to solutions of [(Bn-tpen)Mn^II^]^2+^ complex. In MeCN there is a change from yellow to greenish immediately after the addition of *tert*-butyl hydroperoxide, and next to yellow–brown within 24 h, in MeOH solutions—from yellow to brown within 24 h.

The reaction has also been monitored by UV–vis spectroscopy (Fig. S-1). The complex of [(Bn-tpen)Mn^II^]^2+^ shows the absorption peak at *λ* = 262 nm. The addition of 2 mM *t*-BuOOH to 2 × 10^−6^ mol% [(Bn-tpen)Mn^II^]^2+^ and 40 mM cyclohexene causes: (a) only slight increase in the absorption band at *λ* = 262 nm both in acetonitrile and methanol, (b) the formation of the new peaks at *λ* ≈ 232 nm in both solvents, the shift to higher energies could suggest the lengthening of the Mn–O bond, (c) the appearance of a broad band at approximately 300 nm in acetonitrile solutions, this band grows with reaction time, (d) the decrease of the band at *λ* = 380 nm with reaction time (Fig. S-1a). One isosbestic point (*λ* = 365 nm) is visible in the spectra (Fig. S-1a insert). When *t*-BuOOH was replaced with dioxygen the increase of the band at *λ* ≈ 300 nm was also observed in both solvents. It follows from this experiments that the combination of the catalyst, *t*-BuOOH and the substrate is necessary to form an active complex, which carries on the oxidation process. The [(Bn-tpen)Mn^III^- OO(*t*-Bu)]^+^ is formed, and then the active complex reacts with the substrate. Those behaviour was also observed previously (Matsushita et al. 1999; Woitiski et al. 2004; Szczepanik and Sobkowiak 2008).

**Fig. 6:**
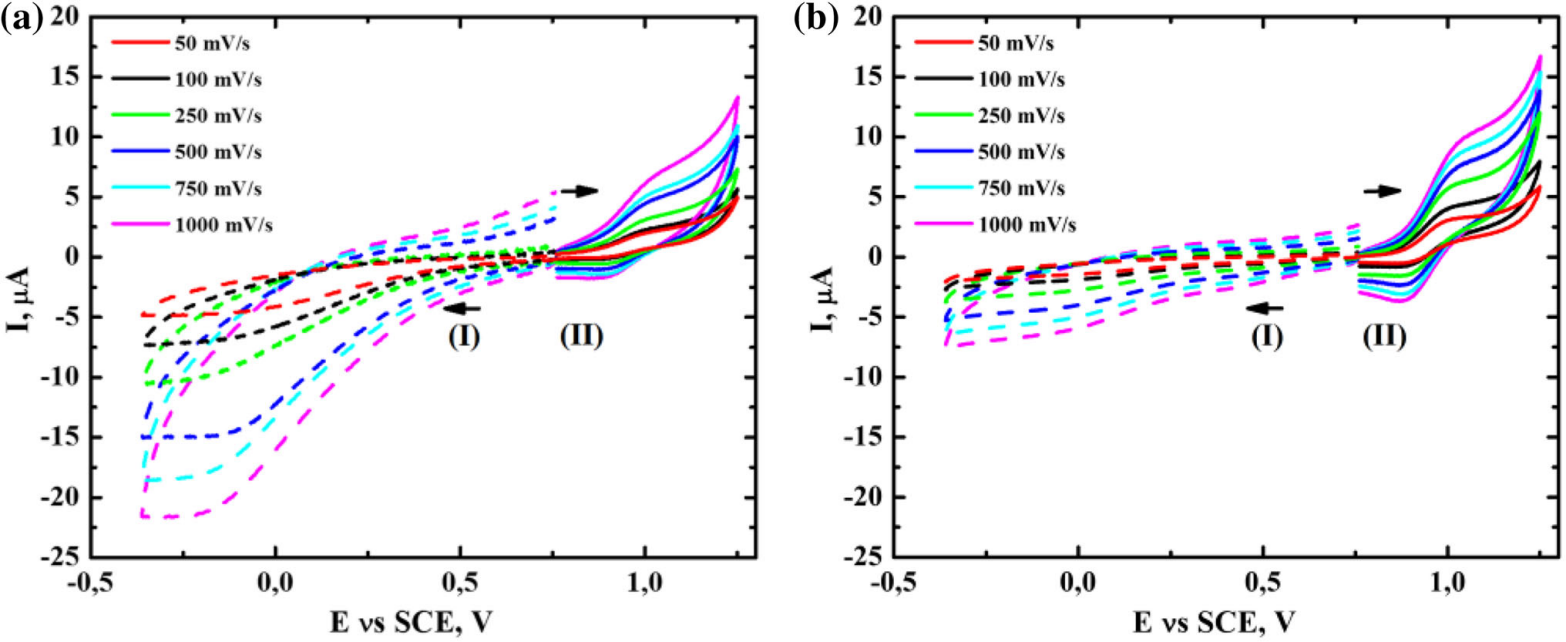
Cyclic-voltammograms in acetonitrile [0.1 M (Et_4_N)ClO_4_] for **a** the mixture of 1 × 10^−3^ mol% [(Bn-tpen)Mn^II^]^2+^, 50 mM *t*-BuOOH and 1 M cyclohexene immediately after mixing, **b** the same as **a** but after 24 h under Ar atmosphere; (*I*) cathodic scan was recorded first, (*II*) anodic scan was recorded first. Scan rate: 0.05; 0.1; 0.25; 0.5; 0.75; 1 V s^−1^, GCE (0.008 cm^2^); SCE vs. NHE, +0.242 V

The cyclic voltammetry for the system containing 1 × 10^−3^ mol % [(Bn-tpen)Mn^II^]^2+^, 50 mM *t*-BuOOH and 1 M cyclohexene in acetonitrile performed immediately after mixing the reactants, shows that in the first cathodic scan (Fig. 6a I-dashed line), the reduction peak at the potential of approximately 0.0 V (vs. SCE) is present, whereas in the first anodic scan (Fig. 6a II-solid line) there is a minute oxidation peak of Mn(II)-complex at 1.1 V (vs. SCE). In separate experiments we confirmed that the addition of a three-fold excess of *tert*-butyl hydroperoxide over the complex causes lowering of the oxidation peak of [(Bn-tpen)Mn^II^]^2+^ at a potential of 1.1 V (vs. SCE) by a factor of 3 (Fig. S-2—compare the curves a and d). After 24 h of reaction under argon atmosphere, the system was analysed again. There were no peaks in the first cathodic scan (Fig. 6b I) whereas oxidation of Mn(II)-complex was visible in the first anodic scan (Fig. 6b II) with some degree of reversibility. The results indicate that in the course of the oxidation of cyclohexene, [(Bn-tpen)Mn^II^]^2+^ is initially oxidized by *t*-BuOOH to Mn(III)-complex, and then Mn(III)-complex is reduced by cyclohexene to [(Bn-tpen)Mn^II^]^2+^, therefore, the complex of Mn(II) ions catalyses the reaction of oxidation of *c*-C_6_H_10_. Adapting the procedure described in (Uruş et al. 2017) the measurements at different scan rates were done (Fig. 6, and Fig. S-3) and the linearity of the dependence $\left| I \right|\; = \; {\text{f}}(\nu^{ 1/ 2} )$ was examined. The linear dependencies observed at potentials −0.1, 0.9 and 1 V (Fig. S-3) indicate that the processes are controlled by the rate of diffusion of the electroactive substance into the electrode surfaces. The process registered at −0.1 V is irreversible one.

**Fig. 7:**
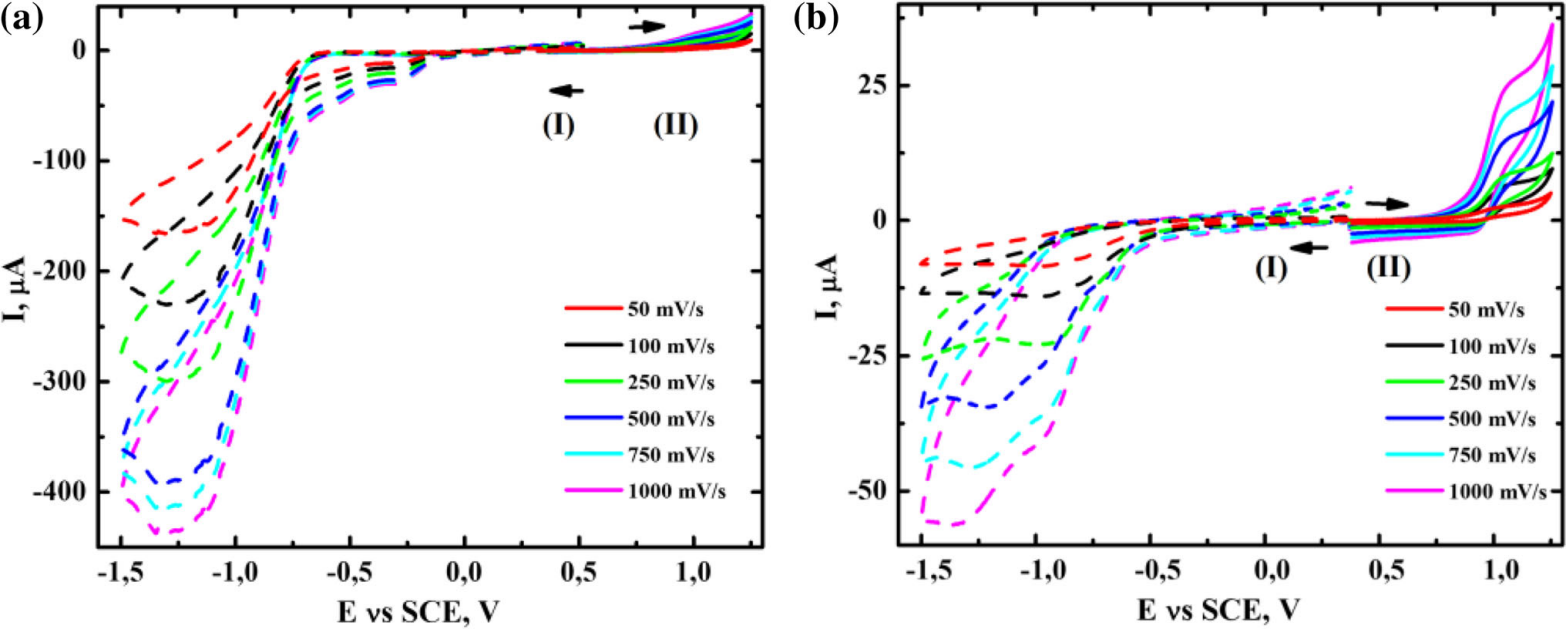
Cyclic-voltammograms in acetonitrile [0.1 M (Et_4_N)ClO_4_] for **a** the mixture of 1 × 10^−3^ mol% [(Bn-tpen)Mn^II^]^2+^, 50 mM HOOH and 1 M cyclohexene immediately after mixing, **b** the same as **a** but after 24 h under Ar atmosphere; (*I*) cathodic scan was recorded first, (*II*) anodic scan was recorded first. Scan rate: 0.05; 0.1; 0.25; 0.5; 0.75; 1 V s^−1^, GCE (0.008 cm^2^); SCE vs. NHE, +0.242 V

When hydrogen peroxide was used instead of *tert*-butyl hydroperoxide as an oxidant, the peak corresponding to dioxygen reduction was registered at −1.25 V (Fig. 7a I), and the height of the peak decreased after 24 h (Fig. 7b I). The non-linearity of the dependence $\left| I \right|\; = \; {\text{f}}(\nu^{ 1/ 2} )$ observed at −1.25 V (Fig. S-4) indicates that the process is not purely diffusive and can be complicated, e.g., by the occurrence of chemical reactions, therefore, the process is irreversible. In turn the processes related to the peaks at 0.9 and 1 V (Fig. S-4) are diffusion controlled.

During [(Bn-tpen)Mn^II^]^2+^-catalyzed oxidation of *c*-C_6_H_10_ by HOOH, hydrogen peroxide is decomposed to O_2_ and H_2_O. The lowering of the dioxygen reduction peak after 24 h (Fig. 7a, b) stems from its diffusion into the surroundings during the reaction. The kinetics of dioxygen evolution from the system containing 5 × 10^−3^ mol% [(Bn-tpen)Mn^II^]^2+^ and 50 mM HOOH was studied (Fig. S-5a). We noticed that after the short induction period hydrogen peroxide starts to decompose in the presence of Mn(II)-complex and for every 1 mol of HOOH approximately 0.3 mol of dioxygen are evolved during 3 h of the reaction (theoretically 1 mol of HOOH can decompose evolving 0.5 mol of O_2_). In the absence of [(Bn-tpen)Mn^II^]^2+^ decomposition of hydrogen peroxide does not occur.

For the oxidation experiments performed under air or argon atmospheres, no important differences between cyclic-voltammetric curves were observed over 24 h, apart from the presence of the small dioxygen reduction peak in the voltammogram for air-containing system.

It has been suggested that iron complexes, such as [(N4Py)Fe^II^]^2+^ and [(Bn-tpen)Fe^II^]^2+^ show different electrochemical behaviour in methanol in comparison to acetonitrile (Hong et al. 2009). However, for the manganese(II) complexes with Bn-tpen, no significant changes in cyclic voltammograms occur when the solvent is changed to MeOH. In methanol, the CV experiment carried out in a solution of the same composition as used in MeCN, i.e., containing 1 × 10^−3^ mol% [(Bn-tpen)Mn^II^]^2+^, 50 mM *t*-BuOOH and 1 M cyclohexene, also allows to conclude, that Mn(III)-complex is reduced by cyclohexene to regenerate an active form of a catalyst—[(Bn-tpen)Mn^II^]^2+^. As shown in Fig. S-6, the reduction peak of Mn(III) complex at +0.1 V (vs. SCE) is present only at the beginning of the reaction. After 24 h all manganese ions are present in the Mn(II) form —no reduction peak in the cathodic scan is observed (Fig. S-6b). The linearity of the dependence $\left| I \right|\; = \; {\text{f}}(\nu^{ 1/ 2} )$ was also examined, electrochemical processes occurred at potentials 0.1, 0.9, 1 V (Fig. S-7) are controlled by the diffusion. In MeOH the reduction peak of Mn(III) ions is shifted to more positive potentials, compared to MeCN. In this environment HOOH is also decomposed in the presence of [(Bn-tpen)Mn^II^]^2+^. The rate of decomposition is quite slow, and after 3 h of the reaction from 1 mol of HOOH, 0.23 mol of dioxygen was obtained (Fig. S-5b). No noticeable changes in cyclic voltammograms were visible for [(Bn-tpen)Mn^II^]^2+^-catalysed oxidations of cyclohexene in MeOH, when using air as an oxidant or in Ar atmosphere.

According to the literature (Cho et al. 2012; Yoon et al. 2013; Yoon et al. 2014), PhIO which is a strong oxygen donor, introduced to the solutions of [(Bn-tpen)Mn^II^]^2+^ causes formation of [(Bn-tpen)Mn^IV^=O]^2+^ oxo-complexes. In voltammograms we registered for this system, the peak at about −0.1 V corresponding to the reduction of [(Bn-tpen)Mn^IV^=O]^2+^ was observed (Fig. S-8a). Addition of a proton-donating substance (HClO_4_) shifts the reduction peak to more positive potentials (Fig. S-8b), what can be explained by incorporation of H^+^ into the process:

$$[ ( {\text{Bn-tpen)Mn}}^{\text{IV}} {\text{ = O]}}^{{ 2 { + }}} {\text{ + H}}^{ + } {\text{ + e}}^{-} \to {\text{ [(Bn-tpen)Mn}}^{\text{III}} {-}{\text{OH]}}^{{ 2 { + }}}$$

Unfortunately the yields of cyclohexene oxidations in MeCN, as well as in MeOH, catalysed by [(Bn-tpen)Mn^IV^=O]^2+^ were rather low.

**Fig. 8:**
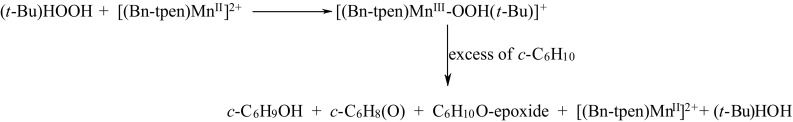
The reactions sequence proposed for oxidation of cyclohexene by hydrogen peroxide (HOOH) or *tert*-butyl hydroperoxide (*t*-BuOOH) catalyzed by [(Bn-tpen)Mn^II^]^2+^

The results presented show that the combination of [(Bn-tpen)Mn^II^]^2+^ and oxygen or hydroperoxides causes the oxidation of cyclohexene to 2-cyclohexen-1-one and 2-cyclohexen-1-ol as the main products, cyclohexene oxide is formed in small amounts. The product profiles are dependent on the solvent used for the experiments. In MeCN solutions, dioxygen (*p*_O2_ = 1 atm) performs better than air or hydroperoxides as an oxidant of cyclohexene in the reactions catalyzed by [(Bn-tpen)Mn^II^]^2+^ (Fig. 4a, Table S-1, S-2, S-3). It is characteristic that the use of HOOH or *t*-BuOOH as an oxidant results in the formation of 2-cyclohexen-1-ol as a main product. The reaction discussed, is not selective one; however, the catalyst applied for the process is stable during the reaction span. The main oxidation product in MeOH is 2-cyclohexen-1-one; however, its concentrations are quite small (Fig. 4b, Table S-1, S-4). Alcohol and epoxide were generally not detected. It follows from the experiments conducted, that the proper combination of the catalyst, substrate and oxygen donor (like dioxygen, or hydroperoxides with reactions $2 {\text{HOOH}}\mathop{ \xrightarrow{{\rm{Mn - Catalyst}}}}{\text{O}}_{ 2} \;{ + }\; 2 {\text{H}}_{ 2} {\text{O}}$ and $2t{\text{-BuOOH}}\mathop{ \xrightarrow{{\rm{Mn - Catalyst}}}}{\text{O}}_{ 2} \;{ + }\;2t{\text{-BuOH}}$) is necessary to enable the formation of an active form of a catalyst. The tentative mechanism of the process can be proposed (Fig. 8).

The reactions of [(Bn-tpen)Mn^II^]^2+^ with HOOH or *t*-BuOOH may be accompanied by decay of the catalysts, which explains poor performance of this systems in cyclohexene oxidations (Table S-3, S-4). To verify this hypothesis, the reactions of [(Bn-tpen)Mn^II^]^2+^/air system with cyclohexene were conducted in the presence of water—the main product of HOOH decay (Table S-5). The concentrations of the products, as we expected, were significantly lower than those obtained in the corresponding non-aqueous systems. Therefore, it seems obvious, that the catalyst molecule is deactivated in the presence of strongly interacting molecules, like water or alcohol. Probably, the more polar alcohol hydroxyl group better coordinates manganese(II) ion in a free coordination site than –C≡N group, and consequently the formation of oxygen adducts is hindered due to competition effect.

As demonstrated, the voltammetric measurements show that in acetonitrile as well as in methanol solutions [(Bn-tpen)Mn^II^]^2+^complex is initially oxidized by *t*-BuOOH to produce Mn(III)-complex, which is reduced back by cyclohexene to complexed Mn(II) ions. The [(Bn-tpen)Mn^II^]^2+^ is an active catalyst of *c*-C_6_H_10_ oxidation. The reduction process of transition metal ion(III) complex with 2,2′-bipyridyl ligand by cyclohexene substrate during the reaction of its oxidation was observed for iron(III) (Sobkowiak et al. 2000) and manganese(III) (Matsushita et al. 1999; Szczepanik and Sobkowiak 2008).

Taking into account the literature (Shul’pin et al. 2001; Woitiski et al. 2004; Szczepanik and Sobkowiak 2008; Shul’pin et al. 2010), all the described results, the reaction of molecular oxygen with cyclohexene and [(Bn-tpen)Mn^II^]^2+^ gives the hypothetical adduct ([(Bn-tpen)Mn^IV^(OOH)(C_6_H_9_)]^2+^) which in the presence of an excess of dioxygen and cyclohexene leads to the very reactive adduct ([(Bn-tpen)Mn^IV^(OH)(OOC_6_H_9_)]^2+^). This active species may undergo further reactions involving formation of ketone, alcohol, and/or epoxide.

## Conclusions

In this work, we demonstrated how the selection of the proper solvent determines the adequate selectivity and productivity of the reaction of cyclohexene oxidation. Ketone is usually the dominant oxidation product in more polar MeOH for all oxidants in [(Bn-tpen)Mn^II^]^2+^-catalysed oxidations. However, in MeCN when we choose dioxygen as an oxidant, ketone and alcohol (mostly in the ratio 2:1) are formed, but for hydroperoxides used in the same role, alcohol is the main oxidation product and the amounts of respective ketone and epoxide arise too.

Using electrochemical methods, we successfully confirmed formation of the [(Bn-tpen)Mn^III^–OH]^2+^ and [(Bn-tpen)Mn^IV^=O]^2+^complexes. This appears to be the first report where it was found that in acetonitrile, as well as in methanol solutions, [(Bn-tpen)Mn^II^]^2+^ complex is initially oxidized by *t*-BuOOH to produce Mn(III)-complex, which is reduced back by cyclohexene to complexed Mn(II) ions. The [(Bn-tpen)Mn^II^]^2+^ is an active catalyst of *c*-C_6_H_10_ oxidation.

The results presented indicate, that the oxidation of cyclohexene by dioxygen catalyzed by [(Bn-tpen)Mn^II^]^2+^ complexes can enable to produce valuable products. The reaction is not selective, but the catalyst is stable over extended period of time. The information concerning the reactions sequence we provided can contribute to better understanding of action of enzymes and oxygen toxicity.

## Electronic supplementary material

Below is the link to the electronic supplementary material.

Supplementary material 1 (DOCX 18346 kb)
