# Supplementary material for: Manganese(II) complexes with Bn-tpen as powerful catalysts of cyclohexene oxidation
Source: Chem Zvesti. 2017 May 26;71(11):2085–93. doi: 10.1007/s11696-017-0201-0 (PMC5655609; doi:10.1007/s11696-017-0201-0)
Supplement: Supplementary file 1 — Supplementary material 1 (DOCX 18346 kb) [file 11696_2017_201_MOESM1_ESM.docx]

**Manganese(II) Complexes with Bn-tpen as Powerful Catalysts of Cyclohexene Oxidation**

# Katarzyna Rydel-Ciszek*, Maria Charczuk, Tomasz Pacześniak, Paweł Chmielarz

# *Department of Physical Chemistry, Faculty of Chemistry, Rzeszów University of Technology, 35-959 Rzeszów, Poland*

# * Corresponding author: e-mail: [kasiar@prz.edu.pl](mailto:kasiar@prz.edu.pl)

# Supplementary Information

**Table S-1.** Oxidation of 1.0 M cyclohexene by dioxygen catalyzed by [(Bn-tpen)Mn^II^]^2+^. Reaction time: 24 h.

| Solvent |  |  | MeCN as a solvent | | | |  | MeOH as a solvent | | | |
| --- | --- | --- | --- | --- | --- | --- | --- | --- | --- | --- | --- |
| Catalyst Concen-tration, mol% | Oxidant |  | Ketone,  mM | Alcohol,  mM | Epoxide,  mM | TON |  | Ketone,  mM | Alcohol,  mM | Epoxide,  mM | TON |
| 2.5∙10^-4^ | O_2_ |  | 84.6 | 54.4 | 7.0 | 292.0 |  | 39.1 | 1.1 | 0.8 | 82.0 |
| 2.5∙10^-4^ | Air |  | 61.4 | 35.7 | 4.0 | 202.2 |  | 38.4 | 0.6 | 1.0 | 80.0 |
| 5∙10^-4^ | O_2_ |  | 119.3 | 58.5 | 9.7 | 187.5 |  | 27.1 | 1.0 | 0.9 | 29.0 |
| 5∙10^-4^ | Air |  | 97.0 | 27.9 | 9.1 | 134.0 |  | 34.2 | 5.1 | 0.8 | 40.1 |
| 1.25∙10^-3^ | O_2_ |  | 69.9 | 19.1 | 7.3 | 38.5 |  | 51.3 | 13.3 | 1.0 | 26.2 |
| 1.25∙10^-3^ | Air |  | 40.0 | 11.3 | 5.0 | 22.5 |  | 47.7 | 7.7 | 1.0 | 22.6 |
| 2.5∙10^-3^ | O_2_ |  | 86.1 | 33.1 | 9.1 | 25.7 |  | 44.9 | 7.4 | 0.5 | 10.5 |
| 2.5∙10^-3^ | Air |  | 13.3 | 3.4 | 2.7 | 3.9 |  | 46.1 | 0.0 | 0.7 | 9.4 |
| 3.75∙10^-3^ | O_2_ |  | 17.2 | 16.0 | 2.0 | 3.3 |  | 55.2 | 10.6 | 0.9 | 8.9 |
| 3.75∙10^-3^ | Air |  | 12.5 | 1.7 | 0.4 | 1.9 |  | 44.5 | 0.0 | 0.6 | 6.0 |
| 5∙10^-3^ | O_2_ |  | 26.9 | 10.4 | 1.3 | 3.8 |  | 41.3 | 2.0 | 0.5 | 4.4 |
| 5∙10^-3^ | Air |  | 9.7 | 16.6 | 2.6 | 2.9 |  | 45.6 | 0.0 | 0.7 | 4.6 |

TON- product molecules per catalyst molecule, Ketone – (2-cyclohexen-1-one), Alcohol – (2-cyclohexen-1-ol), Epoxide – (cyclohexene oxide)

**Table S-2.** Oxidation of cyclohexene by dioxygen catalyzed by 2.5∙10^-4^mol% [(Bn‑tpen)Mn^II^]^2+^. Reaction time: 24 h.

| Substrate Concentration, M | Oxidant | Ketone,  mM | Alcohol,  mM | Epoxide,  mM | TON |
| --- | --- | --- | --- | --- | --- |
| 0.5 | O_2_^(a)^ | 40.4 | 6.1 | 2.6 | 98.2 |
| 0.5 | Air^(b)^ | 24.0 | 0.5 | 0.8 | 50.6 |
| 1 | O_2_^(a)^ | 84.6 | 54.4 | 7.0 | 292.0 |
| 1 | Air^(b)^ | 39.1 | 1.1 | 0.8 | 82.0 |
| 2 | O_2_^(a)^ | 87.8 | 84.6 | 8.7 | 362.2 |
| 2 | Air^(b)^ | 92.0 | 24.6 | 1.8 | 236.8 |
| 3 | O_2_^(a)^ | 145.1 | 138.8 | 22.7 | 613.2 |
| 3 | Air^(b)^ | 125.0 | 54.0 | 8.2 | 374.4 |
| 4 | O_2_^(a)^ | 196.4 | 146.6 | 15.1 | 716.2 |
| 4 | Air^(b)^ | 146.3 | 77.1 | 21.3 | 489.4 |

TON - product molecules per catalyst molecule

Ketone – (2-cyclohexen-1-one), Alcohol – (2-cyclohexen-1-ol), Epoxide – (cyclohexene oxide)

Solvent: (a) – MeCN, (b) – MeOH

**Table S-3.** Oxidation of 1.0 M cyclohexene by hydroperoxides catalyzed by [(Bn-tpen)Mn^II^]^2+^ in acetonitrile. Reaction time: 24 h.

|  |  |  | HOOH as oxidant | | | |  | *t*-BuOOH as oxidant | | | |
| --- | --- | --- | --- | --- | --- | --- | --- | --- | --- | --- | --- |
| Catalyst Concen-tration,  mol% | Oxidant Concen-tration, mM |  | Ketone,  mM | Alcohol,  mM | Epoxide,  mM | TON |  | Ketone,  mM | Alcohol,  mM | Epoxide,  mM | TON |
| 2.5∙10^-4^ | 25 |  | 5.1 | 10.5 | 2.5 | 36.2 |  | 5.2 | 8.6 | 2.2 | 32.0 |
| 2.5∙10^-4^ | 50 |  | 7.9 | 12.5 | 4.9 | 50.6 |  | 6.3 | 10.3 | 3.6 | 40.4 |
| 2.5∙10^-4^ | 100 |  | 1.3 | 6.6 | 3.8 | 23.4 |  | 5.9 | 10.0 | 3.3 | 38.4 |
| 5∙10^-4^ | 25 |  | 8.7 | 13.6 | 4.8 | 27.1 |  | 6.7 | 11.1 | 3.5 | 21.3 |
| 5∙10^-4^ | 50 |  | 8.9 | 12.5 | 2.4 | 23.8 |  | 4.0 | 10.1 | 1.9 | 16.0 |
| 5∙10^-4^ | 100 |  | 12.5 | 18.0 | 8.0 | 38.5 |  | 4.8 | 10.0 | 1.9 | 16.7 |
| 2.5∙10^-3^ | 25 |  | 3.7 | 7.5 | 1.0 | 2.4 |  | 3.9 | 10.4 | 0.0 | 2.9 |
| 2.5∙10^-3^ | 50 |  | 7.3 | 11.1 | 1.6 | 4.0 |  | 16.9 | 24.1 | 3.1 | 8.8 |
| 2.5∙10^-3^ | 100 |  | 4.8 | 9.9 | 1.3 | 3.2 |  | 22.4 | 27.8 | 3.1 | 10.7 |

TON - product molecules per catalyst molecule

Ketone – (2-cyclohexen-1-one), Alcohol – (2-cyclohexen-1-ol), Epoxide – (cyclohexene oxide)

**Table S-4.** Oxidation of 1.0 M cyclohexene by hydroperoxides catalyzed by [(Bn-tpen)Mn^II^]^2+^ in methanol. Reaction time: 24 h.

|  |  |  | HOOH as oxidant | | | |  | *t*-BuOOH as oxidant | | | |
| --- | --- | --- | --- | --- | --- | --- | --- | --- | --- | --- | --- |
| Catalyst Concen-tration,  mol% | Oxidant Concen-tration, mM |  | Ketone,  mM | Alcohol,  mM | Epoxide,  mM | TON |  | Ketone,  mM | Alcohol,  mM | Epoxide,  mM | TON |
| 2.5∙10^-4^ | 25 |  | 0.9 | 0.0 | 0.0 | 2 |  | 4.7 | 0.0 | 0.1 | 10 |
| 2.5∙10^-4^ | 50 |  | 3.1 | 0.0 | 0.0 | 6 |  | 5.9 | 0.2 | 0.3 | 13 |
| 2.5∙10^-4^ | 100 |  | 3.5 | 0.0 | 0.0 | 7 |  | 5.8 | 0.2 | 0.1 | 12 |
| 5∙10^-4^ | 25 |  | 2.3 | 0.0 | 0.0 | 2 |  | 8.5 | 0.8 | 0.1 | 9 |
| 5∙10^-4^ | 50 |  | 3.3 | 0.0 | 0.0 | 3 |  | 7.9 | 0.2 | 0.0 | 8 |
| 5∙10^-4^ | 100 |  | 3.6 | 0.0 | 0.0 | 4 |  | 9.1 | 0.5 | 0.0 | 10 |
| 2.5∙10^-3^ | 25 |  | 5.5 | 0.0 | 0.0 | 1 |  | 5.6 | 1.2 | 0.0 | 1 |
| 2.5∙10^-3^ | 50 |  | 8.3 | 0.0 | 0.0 | 2 |  | 7.4 | 0.0 | 0.0 | 1 |
| 2.5∙10^-3^ | 100 |  | 4.3 | 0.0 | 0.0 | 1 |  | 11.5 | 1.5 | 0.0 | 3 |

TON - product molecules per catalyst molecule

Ketone – (2-cyclohexen-1-one), Alcohol – (2-cyclohexen-1-ol), Epoxide – (cyclohexene oxide)


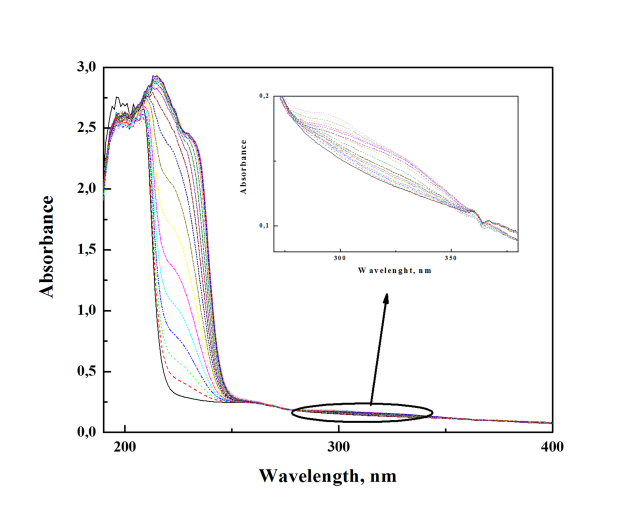

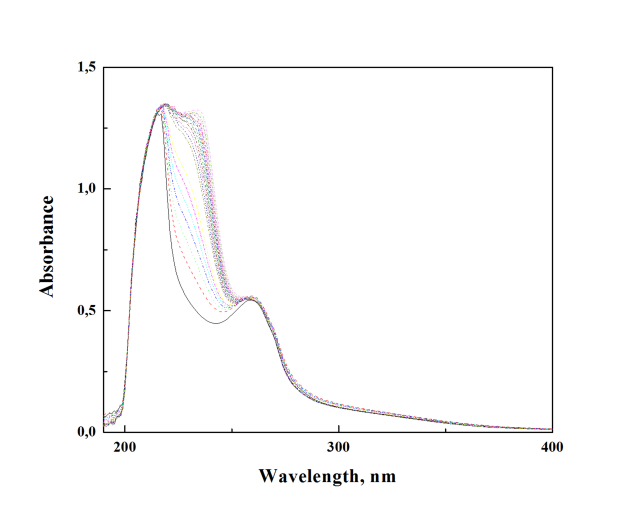


a) b)

**Fig. S-1.** UV-vis spectra over time for the mixture of 2∙10^-6^mol% [(Bn‑tpen)Mn^II^]^2+^, 2 mM *t*-BuOOH and 40 mM cyclohexene under Ar atmosphere in a) acetonitrile, b) methanol.


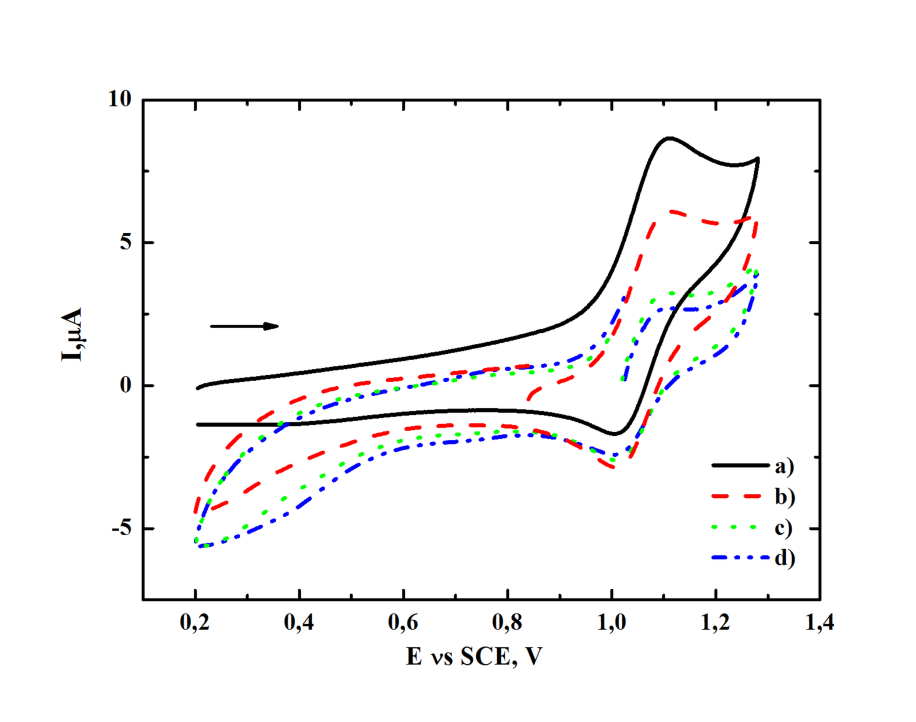


**Fig. S-2.** Cyclic voltammograms for a) 1∙10^-3^ mol% [(Bn-tpen)Mn^II^]^2+^ in MeCN [0.1 M (C_4_H_9_)_4_NClO_4_] and after addition of: b) 5 mM, c) 10 mM, d) 15 mM *t*-BuOOH. Scan rate 0.1 V∙s^-1^, GCE (0.008 cm^2^), SCE *vs.* NHE +0.242 V.


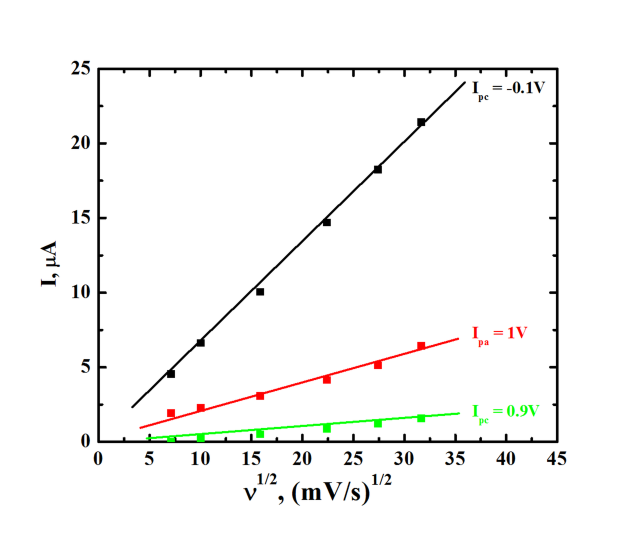

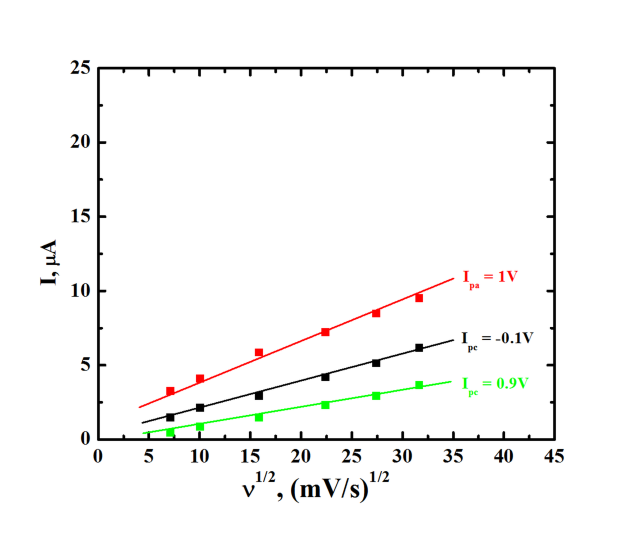


a) b)

**Fig. S-3.** The dependence of the peak currents on square root of scan rate registered in acetonitrile [0.1 M (Et_4_N)ClO_4_] for the mixture of 1∙10^-3^ mol% [(Bn-tpen)Mn^II^]^2+^, 50 mM *t*‑BuOOH and 1 M cyclohexene immediately after mixing a), and b) after 24 h under Ar atmosphere.


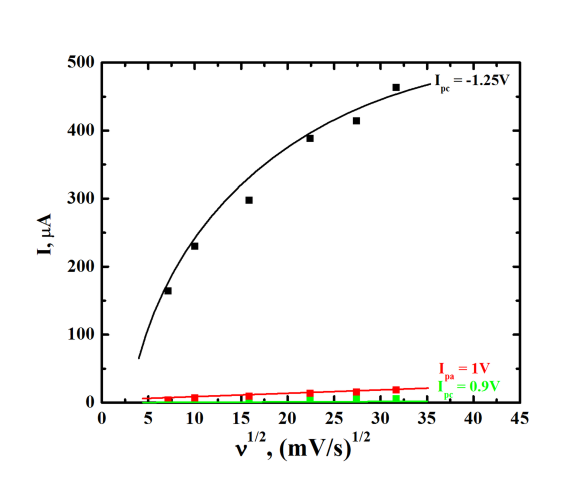

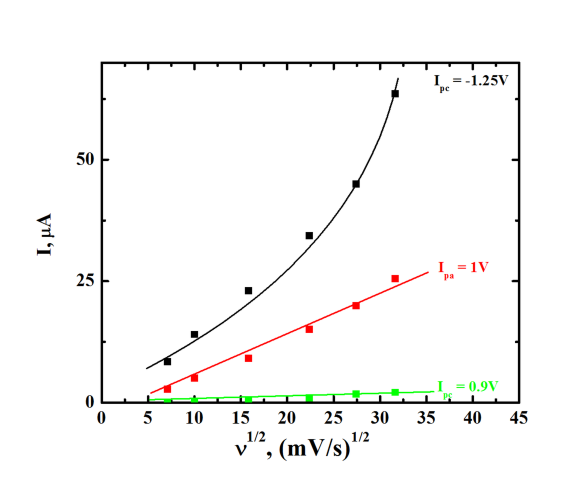


a) b)

**Fig. S-4.** The dependence of the peak currents on square root of scan rate registered in acetonitrile [0.1 M (Et_4_N)ClO_4_] for the mixture of 1∙10^-3^ mol% [(Bn-tpen)Mn^II^]^2+^, 50 mM HOOH and 1 M cyclohexene immediately after mixing a), and b) after 24 h under Ar atmosphere.


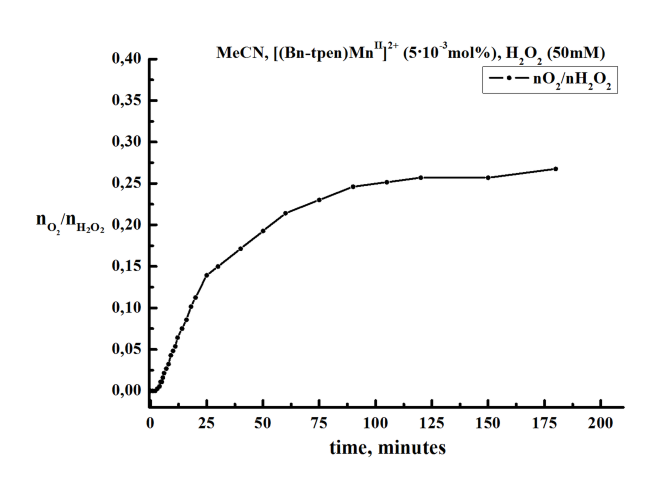

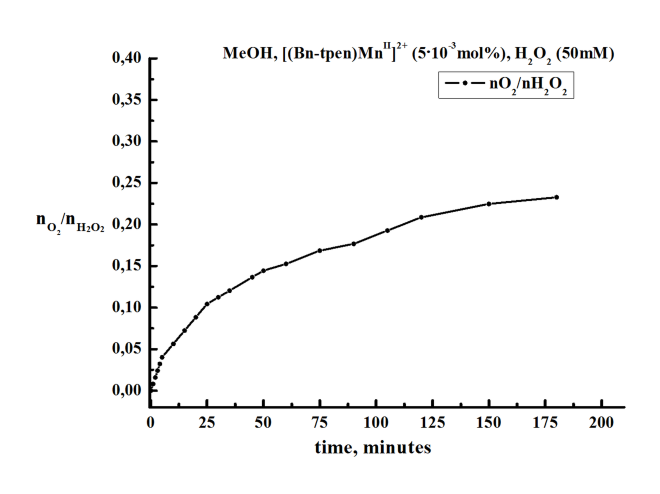


a) b)

**Fig. S-5.** Oxygen evolution during the decomposition of hydrogen peroxide for the mixture of 5∙10^-3^ mol% [(Bn-tpen)Mn^II^]^2+^, 50 mM HOOH in a) acetonitrile, b) methanol, at room temperature (23 ± 1^o^C).

n_O2_/n_H2O2_ is the ratio of oxygen amount evolved to hydrogen peroxide used


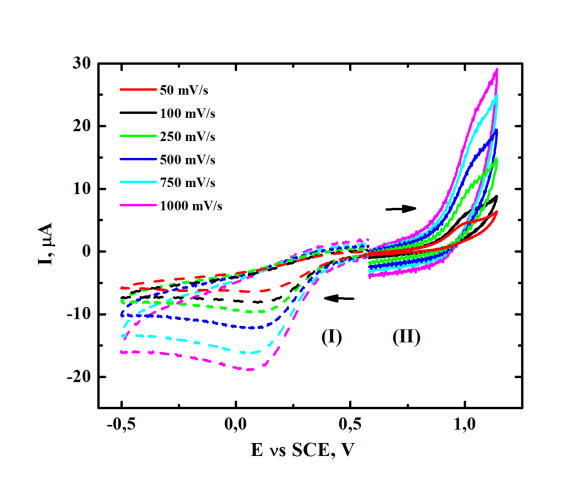

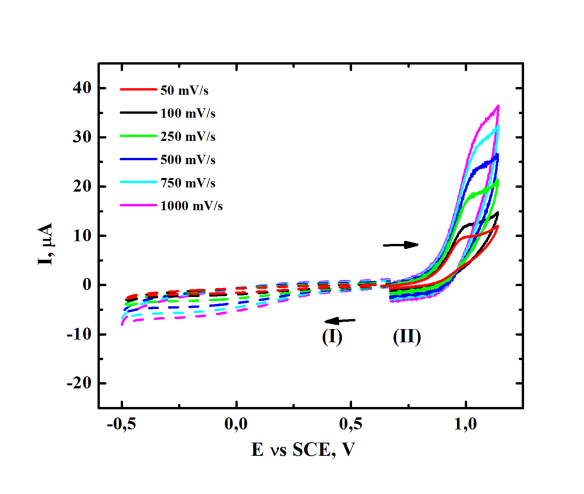


1. b)

**Fig. S-6.** Cyclic-voltammograms in methanol [0.1 M (Et_4_N)BF_4_] for a) the mixture of
1∙10^-3^ mol% [(Bn-tpen)Mn^II^]^2+^, 50 mM *t*-BuOOH and 1 M cyclohexene, a) immediately after mixing b) after 24 h under Ar atmosphere; (I) cathodic scan was recorded first, (II) anodic scan was recorded first. Scan rate: 0.05; 0.1; 0.25; 0.5; 0.75; 1 V∙s^-1^, GCE (0.008 cm^2^); SCE *vs.* NHE, +0.242 V.


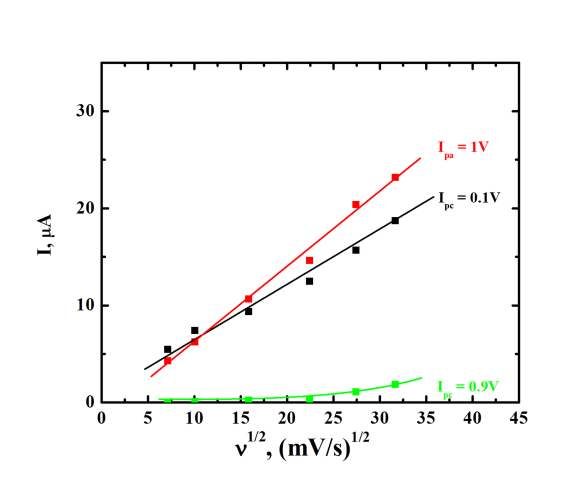

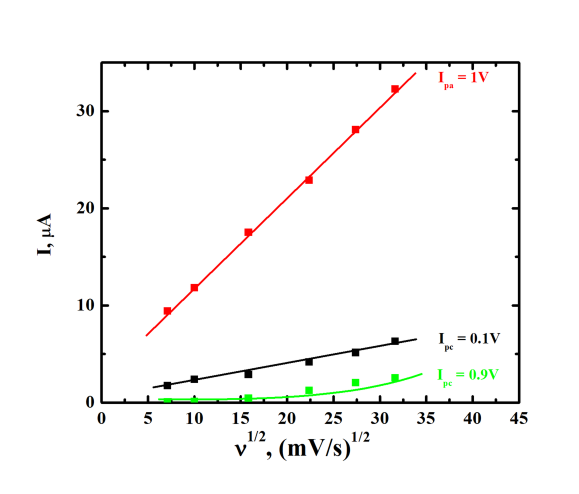


1. b)

**Fig. S-7.** The dependence of the peak currents on square root of scan rate registered in methanol [0.1 M (Et_4_N)BF_4_] for the mixture of 1∙10^-3^ mol% [(Bn-tpen)Mn^II^]^2+^, 50 mM *t*‑BuOOH and 1 M cyclohexene immediately after mixing a), and b) after 24 h under Ar atmosphere.

| 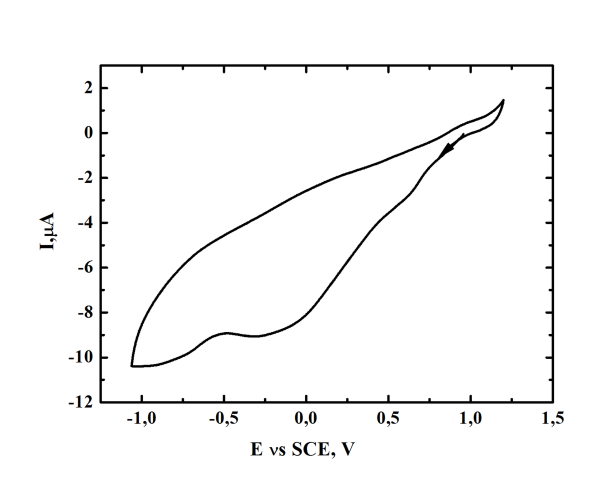 |  |
| --- | --- |
| a) | b) |

**Fig. S-8.** Cyclic voltammograms for 1∙10^-3^ mol% [(Bn-tpen)Mn^II^]^2+^ in MeCN [0.1 M (C_4_H_9_)_4_NClO_4_] after addition: a) of 15 mM PhIO, b) 15 mM HClO_4_ to solution containing [(Bn-tpen)Mn^IV^=O]^2+^ (generated in reaction of manganese(II) complexes with PhIO). Scan rate 0.1 V∙s^-1^, GCE (0.008 cm^2^), SCE *vs.* NHE +0.242 V.

**Table S-5.** Oxidation of 1.0 M cyclohexene by air catalyzed by 2.5∙10^-4^ mol% [(Bn‑tpen)Mn^II^]^2+^ after addition of 1 M H_2_O. Reaction time: 24 h.

| Solvent | Ketone,  mM | Alcohol,  mM | Epoxide,  mM | TON |
| --- | --- | --- | --- | --- |
| MeCN | 5.9 | 3.1 | 1.8 | 21.7 |
| MeOH | 1.3 | 1.2 | 3.1 | 11.1 |

TON - product molecules per catalyst molecule

Ketone – (2-cyclohexen-1-one), Alcohol – (2-cyclohexen-1-ol), Epoxide – (cyclohexene oxide)
